# An Automatic Concrete Crack-Detection Method Fusing Point Clouds and Images Based on Improved Otsu’s Algorithm

**DOI:** 10.3390/s21051581

**Published:** 2021-02-24

**Authors:** Xiaolong Chen, Jian Li, Shuowen Huang, Hao Cui, Peirong Liu, Quan Sun

**Affiliations:** 1 School of Water Conservancy Science & Engineering, Zhengzhou University, Zhengzhou 450001, China; cxlchina001@163.com (X.C.); lpr0531@163.com (P.L.); sunquan@gs.zzu.edu.cn (Q.S.); 2 School of Geo-Science & Technology, Zhengzhou University, Zhengzhou 450001, China; lijian5277@163.com (J.L.); hswzzu@163.com (S.H.)

**Keywords:** concrete crack detection, the fusion of point clouds and images, 3D laser point cloud, Otsu’s algorithm

## Abstract

Cracks are one of the main distresses that occur on concrete surfaces. Traditional methods for detecting cracks based on two-dimensional (2D) images can be hampered by stains, shadows, and other artifacts, while various three-dimensional (3D) crack-detection techniques, using point clouds, are less affected in this regard but are limited by the measurement accuracy of the 3D laser scanner. In this study, we propose an automatic crack-detection method that fuses 3D point clouds and 2D images based on an improved Otsu algorithm, which consists of the following four major procedures. First, a high-precision registration of a depth image projected from 3D point clouds and 2D images is performed. Second, pixel-level image fusion is performed, which fuses the depth and gray information. Third, a rough crack image is obtained from the fusion image using the improved Otsu method. Finally, the connected domain labeling and morphological methods are used to finely extract the cracks. Experimentally, the proposed method was tested at multiple scales and with various types of concrete crack. The results demonstrate that the proposed method can achieve an average precision of 89.0%, recall of 84.8%, and F1 score of 86.7%, performing significantly better than the single image (average F1 score of 67.6%) and single point cloud (average F1 score of 76.0%) methods. Accordingly, the proposed method has high detection accuracy and universality, indicating its wide potential application as an automatic method for concrete-crack detection.

## 1. Introduction

Cracks are one of the main types of distress in concrete surfaces, with a significant impact on the bearing capacity and durability of concrete structures. Therefore, an automatic and accurate method for recognizing them is crucial for the monitoring and maintenance of concrete structures. In the past few years, various technologies have been developed for automatic concrete distress detection. According to the principles of detection, we can divide these into three main categories: 2D image-based detections, 3D laser-scanning methods, and 2D–3D combination techniques. 

Many researchers have proposed various crack-edge detection algorithms based on 2D images, such as Laplacian, Sobel, Canny, and Prewitt [1,2,3,4]. These are effective in detecting cracks with obvious edge-gradient changes but are more sensitive to noise. Otsu et al. [5] proposed an automatic threshold image segmentation method based on the maximum variance (in gray) between classes. This method could calculate an appropriate threshold automatically but performs poorly in the presence of shadows or stains. Nguyen et al. [6] comprehensively considered features of cracks such as the grayscale values, texture, profile shape, and other features, achieving more accurate crack detection. A method based on deep learning has also been used for image crack detection [7,8,9,10,11,12,13], but this method requires many training samples. Although the aforementioned 2D image-based techniques provide various methods for automatic crack detection in specific cases, they are unable to distinguish cracks from artifacts such as illumination and water or oil stains, which is a major problem.

With the recent developments and innovations in hardware, 3D-laser disease-detection technology has become a research hotspot [14,15,16,17,18]. By contrast with the principle of 2D imaging, 3D-laser technology measures the geometric parameters of a target object’s surface and generates abundant high-precision spatial information called a point cloud [19,20], so crack detection is not interfered with by the above factors. There are two main methods for extracting cracks from 3D point clouds. (1) projecting dense 3D point clouds into 2D images and extracting cracks using image-processing techniques [21,22]; and (2) extracting cracks directly from the 3D point clouds [23,24]. Based on the first method, Yang et al. [25] used the reflectance information from point clouds to detect cracks in a tunnel structure through morphology and a Canny edge detection operator, but there were many fractures in the detected cracks. Guan et al. [26] proposed an iterative tensor voting method for the high-precision detection of road cracks. However, this approach was mainly developed to extract the types and locations of pavement cracks rather than the crack widths. Jiang et al. [27] used the inverse distance weighting method to generate point cloud raster images to realize crack detection for post-earthquake buildings. However, this required many shape features to remove false cracks. Based on the second method (direct extraction). Yu et al. [28] used intensity information from the point clouds to directly extract the crack skeleton. Firstly, Otsu’s method was used to extract candidate cracks and, then, a spatial density filter was used to denoise them. Finally, crack skeletons were extracted based on an L1-medial skeleton extraction method. This avoided a time-consuming neighborhood search in dense discrete point clouds and was fast to execute. However, crack points account for only a small proportion of mobile laser scanning data, which made Otsu’s threshold unreliable for segmented cracks. According to the scanning angle and time of the scanner, Zhong et al. [29] established a 2D index for each point cloud to reduce the dimensionality in a non-destructive manner, and reflectance and depth information were then used to extract cracks. Since the density of a 3D point cloud is not high enough, existing 3D methods often make it difficult to detect small cracks.

Considering the complex conditions of concrete surfaces, such as the presence of stains and shadows, it is difficult to achieve high accuracy using a 2D-image method or a 3D point clouds separately. Therefore, some scholars have proposed methods combining 2D and 3D crack extraction [30]. Valença et al. [31] used coordinate information from the point clouds to orthorectify images, solving the problems encountered when applying image-detection technology to detecting cracks in large buildings. However, the method was still based on the image for crack detection and did not make full use of the depth information of point clouds. Based on the Dempster–Shafer (D–S) theory, Huang et al. [32] proposed a new road-crack detection method combining 2D images and 3D-laser-scanning data. Their experimental results showed that this method could improve the accuracy of crack detection and reduce the fallout ratio. However, it only used four structured lights in the same direction to judge whether a crack in the image was real, increasing the possibility of miscalculation.

To overcome the discussed limitations for high precision concrete-surface crack detection based on single 2D images and 3D point clouds methods, an automatic method fusing 3D point clouds and 2D images based on an improved Otsu’s method is proposed in this paper. In this method, depth information from point clouds and gray information is fused at the pixel level; then based on an improved Otsu segmentation method and fine detection of cracks, high-precision crack detection, is achieved. The main innovations of the proposed method in this paper are as follows: (1) a new fusion method of 3D point clouds and 2D image data is proposed; (2) proposing an improved Otsu crack detection method; (3) discussing the application of two different types of 3D laser scanners in the field of crack detection. The rest of the article is organized as follows: the proposed fusion detection method is explained in detail in Section 2, experiments results and discussion are shown in Section 3, and conclusions are given in Section 4. 

## 2. Methods

**Figure 1:**
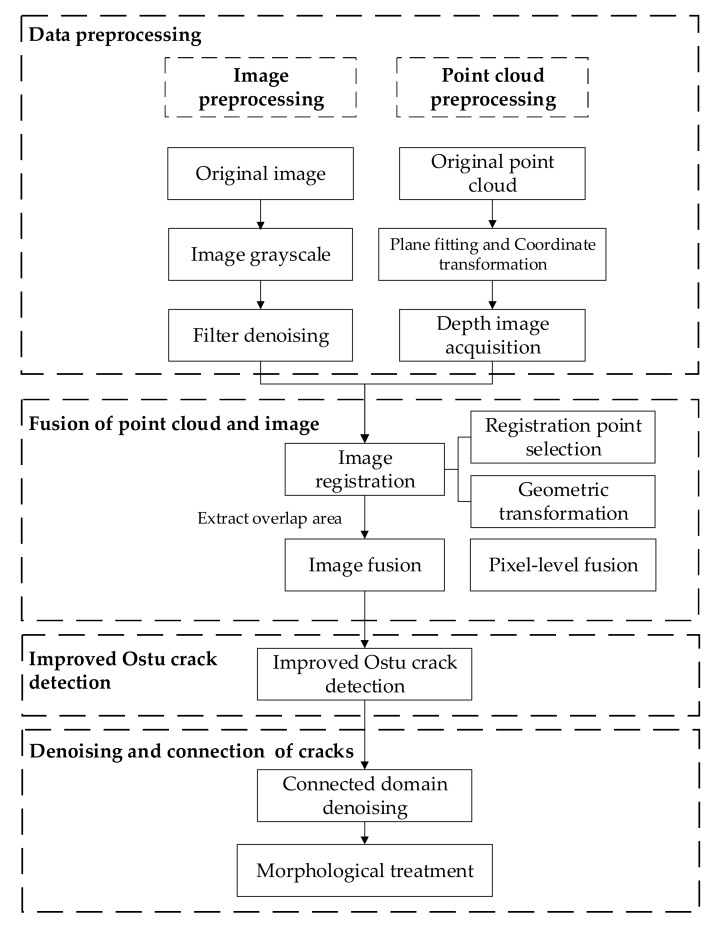
The flowchart of the proposed crack-detection method.

In this study, the overall framework of the proposed method is mainly composed of the following steps: (1) data preprocessing; (2) the fusion of 3D point clouds and 2D images; (3) crack detection based on an improved Otsu’s method; (4) denoising and repair of cracks. Among them, the fusion of 3D point clouds and 2D images and the crack detection based on the improved Otsu’s method are the two most important parts. The former determines the quality of the fusion image and is the basis for high-precision crack detection, while the latter determines the accuracy with which cracks are extracted. A flow chart summarizing the process is presented in Figure 1.

### 2.1. Data Preprocessing

Due to interference from the sensor itself, the environment, and human factors, there is a certain amount of noise in the collected point cloud and image data, so they need to be preprocessed separately. For the images, the three-color component weighted average method is first applied for image graying: the true color image (red, green and blue, RGB) is converted into grayscale by removing a lot of color information that is not good for crack detection, which reduces the image data. Specifically, the 24-bit RGB data ((2^8^)^3^ = 16,777,216 colors) is converted into 8-bit gray-level (2^8^ = 256 levels) data. Image noise is then removed using the median filter to avoid its interference in crack detection, (the median filter was selected after comparing various filtering methods). For the 3D point clouds, the preprocessing mainly includes two steps: (1) the filtering and denoising of the point clouds; and (2) depth-image acquisition. Before fusing the 2D and 3D data, the point clouds need to be reduced into 2D depth images. However, any noise in the original point clouds greatly impacts the quality of the generated depth images; therefore target-area point clouds are extracted from the original point clouds and then denoised with the median filter.

To obtain the depth image, it is necessary to transform the filtered point clouds from the original coordinate system to the new coordinate system with the concrete surface fitting plane as the XOY plane. Firstly, principal component analysis (PCA) is used to fit the point clouds and calculate the parameters of the fitting plane. Next, the rotation and translation matrix of the fitting plane transformed into the XOY plane is calculated. Then, the coordinate of all the point clouds is transformed using the calculated rotation-translation matrix. The points from plane fitting contain crack points, which cause errors in the plane fitting, so the Z coordinate threshold was used to remove the crack point clouds in this study; then, a second plane fitting for the remaining non-crack point clouds was carried out by the PCA method, and the corresponding rotation translation matrix was calculated again. Finally, the rotation and translation matrix was used to transform the coordinates of all the point clouds including the cracked and non-cracked point clouds.

**Figure 2:**
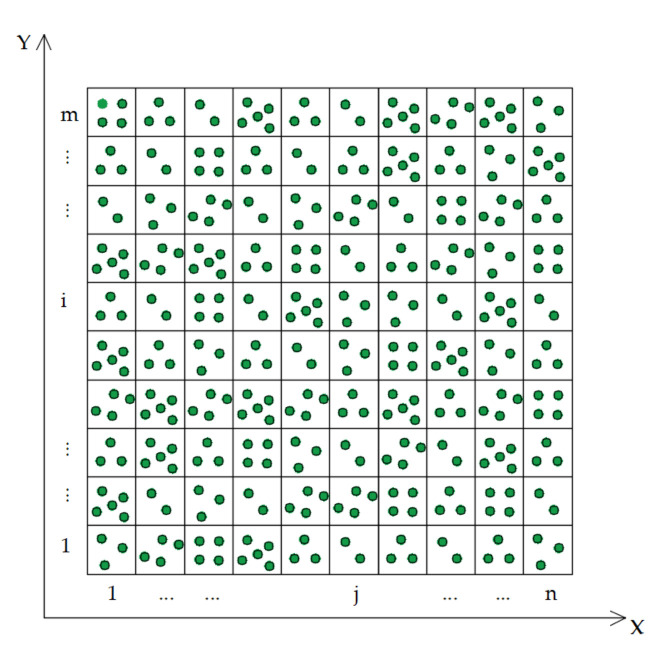
Point clouds projection grid.

The depth of a crack point can be directly expressed by the Z coordinate value of point clouds in the new coordinate system, so point clouds can be projected into depth images as follows:

(1) An image grid of size m×n, as shown in Figure 2 is established, and the edge length of the image pixel is $P_{s}$ (the grid spacing). The relationship between the image size and edge length of the pixel is as follows:
  (1) $$m=\frac{\left[y_{\max}-y_{\min}\right]}{P_{s}},$$
  (2) $$n=\frac{\left[x_{\max}-x_{\min}\right]}{P_{s}},$$
  where $y_{\max}$ is the maximum Y coordinate; $y_{\min}$ is the minimum Y coordinate; $x_{\max}$ is the maximum X coordinate, and $x_{\min}$ is the minimum X coordinate. $P_{s}$ refer to the average point distance of point clouds.
(2) The number of points falling into the grid is calculated, and the average of their coordinate values is taken as the depth.
  (3) $${\bar{z}}_{i,j}=\frac{\sum_{i}^{n}z_{i,j}}{n},$$
  where $z_{i,j}$ is the depth value of row and column of the image grid; ${\bar{z}}_{i,j}$ is the number of points falling into the grid and the average depth value of row and column of the image grid. The reason why the average value is chosen instead of the maximum or minimum value is to avoid error.
(3) The depth is normalized to a 0–255 scale and then the image grid matrix is output as the depth image.
  (4) $$G_{i,j}=\frac{{\bar{z}}_{i,j}-{\bar{z}}_{\min}}{{\bar{z}}_{\max}-{\bar{z}}_{\min}}\times 255,$$
  where ${\bar{z}}_{i,j}$ is the average depth value of row and column of the image grid; ${\bar{z}}_{\max}$ is the maximum average depth value in the image; ${\bar{z}}_{\min}$ is the minimum average depth value in the image.

### 2.2. Fusion of 3D Point Clouds and 2D Gray Images

The fusion of 3D point clouds and 2D images is one of the key aspects of our method. The purpose is to generate a fused image with low noise and prominent cracks by fusing the depth of the point clouds and the image gray information. In this study, the point clouds were projected into depth images, and then, the latter and the 2D gray images were fused by an image processing method mainly consisting of: (1) the registration of the depth and gray image; (2) the fusion of the depth and gray image.

#### 2.2.1. Registration of Depth and Gray Image 

Image registration means overlaying two or more images of the same scene taken at different times, from different viewpoints, and/or by different sensors. It geometrically aligns two images—moving and fixed images. The key is to find the best transformation matrix for maximizing the degree of alignment between the two images, and it mainly involves the calculation of the control points coordinates and geometric transformation. The advantage of the method in this study is that it can not only achieve accurate image registration but also use the target to determine the ground sampling distance (GSD) in the images (the GSD means the actual distance corresponding to the pixel). 

Firstly, we selected circular targets uniformly distributed on the surface of the concrete structure as the control points and calculated their image coordinates The Hough transform circle detection algorithm [33] was used to obtain the center coordinates of the circular target in the 2D images. As for the point clouds, because the 3D laser scanner can directly obtain the precise 3D coordinates of the target center, the corresponding image coordinates can be obtained by calculating the location of the target center point in the depth image.

**Figure 3:**
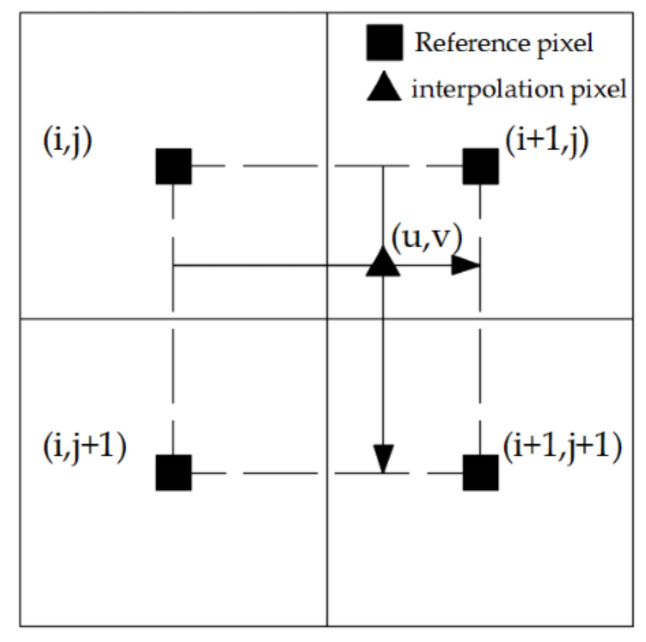
Bilinear interpolation.

After obtaining the precise control point coordinates of the two images, the precise registration of the depth image and the 2D image can be realized through geometric transformation between the two images, which includes spatial geometric transformation and grayscale interpolation. The former defines the pixel distribution of the image in the new coordinate system; the latter assigns the pixel gray level of the image after spatial transformation (grayscale interpolation). This study used a bilinear interpolation method whose principle is shown in Figure 3.

(5) $$I_{2}=g\left(I_{1}\left(f\left(x,y\right)\right)\right),$$

where $I_{1}$ is the reference image; $I_{2}$ is the image to be registered; f represents the geometric transformation of the 2D space; g represents the one-dimensional grayscale transformation.

**Figure 4:**
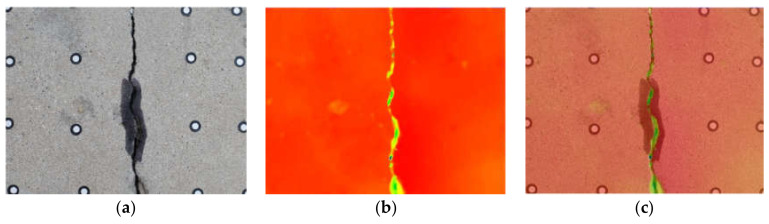
Registration result of depth and gray image. (**a**) image; (**b**) depth image; (**c**) registration result.

To following measures were taken to ensure the accuracy of the registration: (1) a certain number of targets were evenly distributed on the surface of concrete; (2) the center coordinates of the target were obtained by using the circular detection algorithm and fine scan; (3) the two images after registration were overlapped and displayed to ensure that the crack edges completely overlapped. The registration results for the depth and gray image are shown in Figure 4; the depth image is displayed in color in Figure 4b to clarify the registration effect. Figure 4c is the registration effect picture of the two images superimposed. It can be seen from Figure 4c that the crack areas of the two images overlapped well, and the registration result was good.

#### 2.2.2. Fusion of Depth and Gray Image

The fusion of depth and gray images is based on their complementarity for crack detection. For example, in a background area with stain interference, the grayscale of the gray image is smaller than the normal grayscale value, but the grayscale of the depth image is not affected; in an area with muddy landfill or shallow cracks, the grayscale of the depth image is larger than the normal grayscale value, but the grayscale of the gray image is not affected. Harnessing the advantage of the two types of data, and according to the concrete surface background interference and the depth and width of the cracks, we used the weighted-average method of pixel-level fusion to assign different weights to the depth and gray image for data fusion. The fusion image generated by the above steps is a composite of gray information and depth information. Compared with a single image or depth image, it can have reduced background noise and enhanced crack information, providing a solid foundation for subsequent crack detection research.

(6) $$F_{\left(i,j\right)}=\omega_{1}A_{\left(i,j\right)}+\omega_{2}B_{\left(i,j\right)},$$

where A represents the depth image; B represents the image, and (i,j) represents the coordinates of pixels in the image; $\omega_{1}$, $\omega_{2}$ is the weighting factor and usually take $\omega_{1}+\omega_{2}=1$.

**Figure 5:**
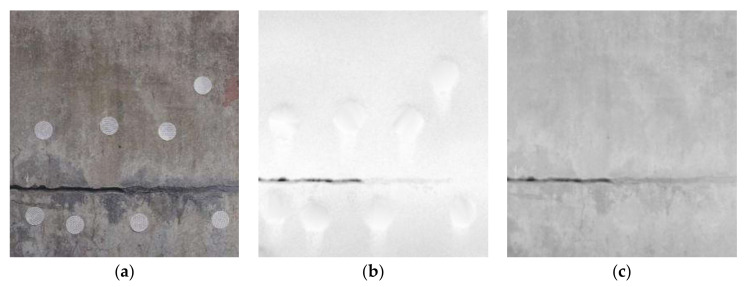
Comparison of different images. (**a**) Original 2D image; (**b**) depth image; (**c**) fusion image.

The comparison of the different images is shown in Figure 5. It can be seen by comparing Figure 5a,c, that the noise from the original image is reduced, and the crack information is enhanced. By comparing Figure 5b,c, it can be observed that the crack information in the fusion image is enhanced, as expected.

### 2.3. Rough Detection of Cracks Based on Improved Otsu’s Method

Otsu’s method is an adaptive threshold crack-detection method that is widely employed. The basic principle is dividing the image into background and target according to a threshold. The best segmentation threshold is the pixel value that maximizes the variance between the two kinds of pixel. Although a fusion image has more advantages for crack detection than a single image or depth image, with the traditional Otsu method, it still presents certain limitations in complex backgrounds and images with unclear background contrasts.

**Figure 6:**
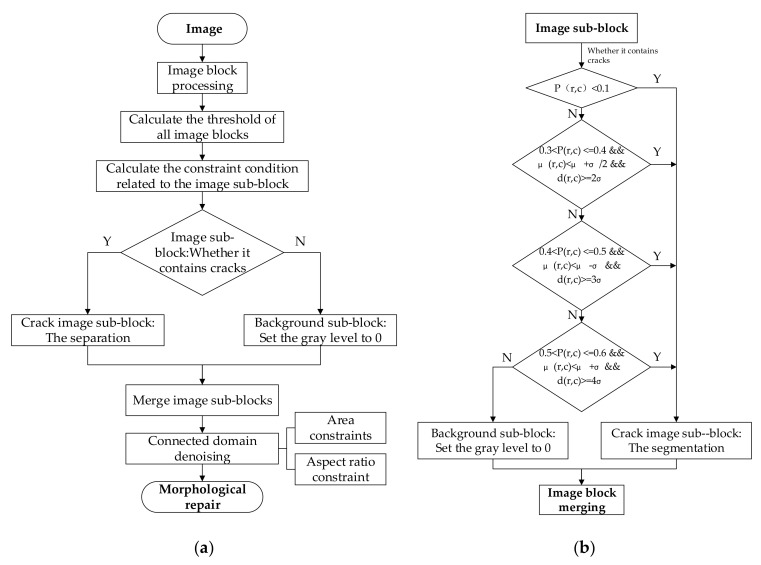
Rough detection of cracks based on improved Otsu’s method. (**a**) Improved overall flow chart of Otsu’s crack detection method; (**b**) classification and segmentation of image sub-blocks.

To overcome the shortcomings of the traditional Otsu method in this regard, this study proposes an improved Otsu crack-detection method that incorporates several constraints for the image-segmentation algorithm, which makes the block-based crack segmentation more accurate. This method first divides the fused image into blocks and calculates the segmentation thresholds for different image sub-blocks. Next, several constraints are introduced to divide the image sub-blocks into the crack and background sub-blocks, and then, the crack sub-blocks are roughly extracted through the steps described above; lastly, all the binary sub-block images are merged, and the fine detection of cracks is realized by denoising and repairing cracks. A flow chart summarizing the improved Otsu crack detection method is shown in Figure 6a, and the specific steps are as follows:

Firstly, the fused image is divided into blocks, and then, the Otsu’s threshold for all the image sub-blocks are calculated. Compared with a single threshold for the whole image, different thresholds for different image sub-blocks can allow the extraction of the cracks from the background with more accuracy.

However, the forced binarization to the background sub-blocks would result in errors. Therefore, we introduced local constraints related to the image sub-blocks such as the pixel ratio $P_{\left(r,c\right)}$, local gray average $\mu_{\left(r,c\right)}$, and the difference in gray average $d_{\left(r,c\right)}$, and global constraints related to the entire image, such as the global gray average μ and global gray standard deviation σ. Based on these constraints, it is judged whether the image sub-block contains cracks. If so, the Otsu threshold segmentation is performed on the image sub-block; otherwise, the grayscale values of all the pixels of the image sub-blocks are set to 0 (background sub-block: no crack). The reason these constraints are introduced is that the proportion of crack pixels in the whole image is small, the difference between the mean gray value of the crack pixels and that of the background pixels is large, and the mean gray value of the image sub-blocks generally fluctuates around the mean gray value of the whole image. In short, the above constraints help distinguish the cracks and background in the image. The specific judgment process for image sub-blocks is shown in Figure 6b, and the specific definitions of the constraints are as follows:

(7) $$P_{\left(r,c\right)}=\frac{n_{\left(r,c\right)}}{N_{\left(r,c\right)}},$$

where $\left(r,c\right)$ is the number of the image sub-block; $n_{\left(r,c\right)}$ is the number of pixels cracked under the threshold $t_{\left(r,c\right)}$ in the image sub-block; $N_{\left(r,c\right)}$ is the number of all pixels in the image sub-block.

(8) $$d_{\left(r,c\right)}=\mu_{1}{}_{\left(r,c\right)}-\mu_{2}{}_{\left(r,c\right)},$$

where $\mu_{1\left(r,c\right)}$ is the gray average of the background of the image sub-block under the threshold $t_{\left(r,c\right)}$; $\mu_{2\left(r,c\right)}$ is the gray average of the crack of the image sub-block under the threshold $t_{\left(r,c\right)}$.

**Figure 7:**
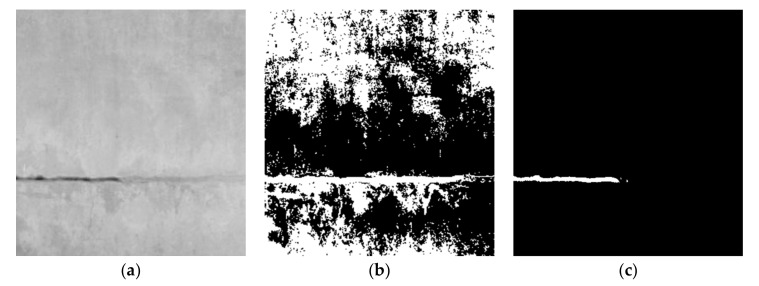
Comparison of crack detection effect of fusion image between traditional Otsu’s method and improved Otsu’s method. (**a**) Fusion image; (**b**) traditional Otsu’s method; (**c**) improved Otsu’s method.

Figure 7 is a comparison between the traditional and improved Otsu methods for crack based on fusion images. Comparing Figure 7b,c, it can be seen that the traditional Otsu method recognizes many background interferences as cracks, resulting in many errors. Meanwhile, the improved Otsu method proposed in this paper can effectively avoid interference from complex backgrounds and accurately detect cracks.

### 2.4. Denoising and Connection of Cracks

**Figure 8:**
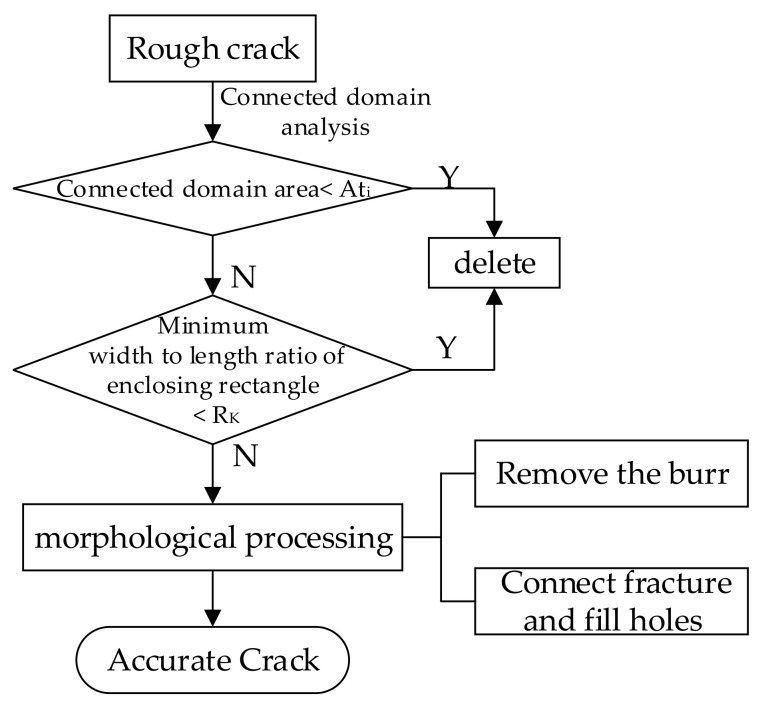
Denoising and connection of cracks.

After the rough crack detection, some crack burrs, noises, and fractures may still exist, which could seriously affect the accuracy. Hence, denoising and connection of cracks are needed to refine the final detection. The cracks in the fusion image mainly present the following two characteristics: (1) generally linear shape; (2) areas are large relative to the noises. Therefore, the connected component labeling method can be used to remove noises based on the shape and area feature constraint of the connected component. Then the morphology method is used to remove the crack burrs and connect the broken cracks. The specific process is shown in Figure 8, and the specific steps are as follows:

Firstly, connected region analysis is performed on the binary image after the implementation of the improved Otsu’s method. First, employing the algorithm used in the connected-region labeling function “bwlabel” in MATLAB, the pixels that conform to the four-connection relation are classified into the same connected domain. For extracting the crack, the connected area and minimum length /width ratio of the circumscribed rectangle of the crack should meet the conditions of Equations (9) and (10).

(9) $$A_{i}\geq At_{i},$$

where $A_{i}$ is the area of the connected region; $At_{i}$ is the area threshold of the connected region conforming to the crack condition.

(10) $$K_{i}=\frac{L_{i}}{W_{i}}\geq R_{K},$$

where $K_{i}$ is the length to width ratio of the connected domain; $L_{i}$ is the length of the connected domain; $W_{i}$ is the width of the connected domain, and $R_{K}$ is the threshold of the length-width ratio of the connected domain satisfying the condition of being a crack.

**Figure 9:**
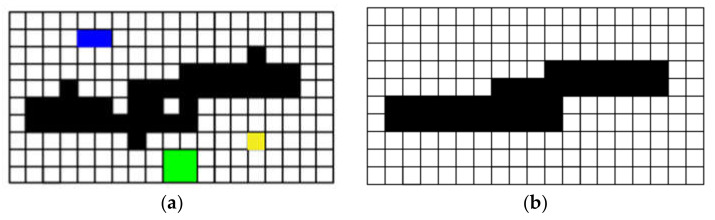
Schematic diagram of the effect before and after denoising and connection of crack. (**a**) Before denoising and connection of crack; (**b**) after denoising and connection of crack.

After the noise is removed based on the above constraints, the open and close operations for morphology can be used to remove the burr and connect the fractured cracks. The crack burrs and isolated noise are removed using the open operation, and the holes in the cracks are filled and the cracks in adjacent areas are connected using the closed operation. Figure 9 is a schematic diagram of the effects before and after the denoising and connection of the crack.

**Figure 10:**
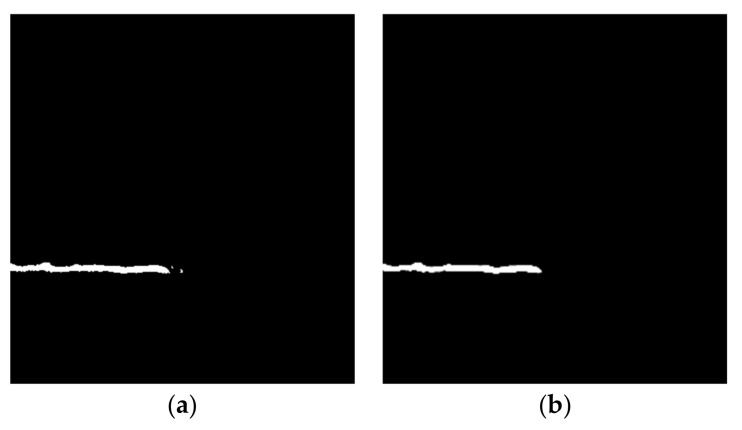
Comparison before and after denoising and connection of cracks: (**a**) before crack denoising and connection of cracks; (**b**) after denoising and connection of cracks.

A comparison of before and after the denoising and connection of cracks is shown in Figure 10, where it can be observed that the noise and burrs of the crack are removed.

## 3. Experimental Results and Analysis

The concrete crack data in this experiment were collected on different pavements and walls at a university covering an area of 3 square kilometers. The cracks include 3D point clouds and 2D-image data. The 3D point clouds were collected by a handscan700 handheld 3D laser scanner and RIEGL VZ-2000 terrestrial 3D laser scanner. The two scanners have different spatial resolutions: the minimum for the handheld scanner is 0.2 mm (the minimum distance between two points), while the point cloud of the terrestrial laser scanner is related to the scanning distance and angular resolution. The shorter the distance, the smaller the angular resolution and the higher the accuracy. To obtain as much 3D information of the crack as possible, we used the highest accuracy that the scanner can achieve, and its corresponding minimum point cloud resolution was 1 mm. The specific technical parameters of the scanner are shown in Table 1 and Table 2.

The 2D images were captured by a camera with a resolution of 7952 × 5304, orthogonally to the concrete surface. The 3D laser scanner and camera were completely separated, each acquiring data separately, so that high-resolution concrete-crack images could be obtained at a close distance. 

Three typical cracks were included: transverse, longitudinal, and inclined. The length and width of the crack concrete area measured by the hand-held 3D laser scanner are about 0.5 m, and the grid spacing of the depth image of the point clouds collected with the handheld 3D laser scanner is 0.5 mm. Therefore, the length and width of the corresponding depth image are about 500 × 500. The length and width of the crack concrete area measured by the terrestrial 3D laser scanner are about 0.6–1.5 m, and the grid spacing of the depth image of the point clouds collected by the terrestrial 3D laser scanner is 1 mm in the experiment. Thus, the length and width of the corresponding depth image were about 600–1500. The relevant parameter settings used for this method are shown in Table 3.

**Figure 11:**
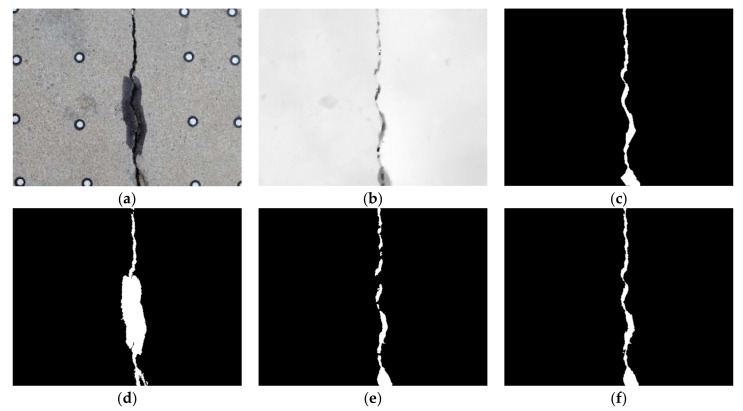
Comparison of crack detection results of different methods based on handheld point clouds: (**a**) image; (**b**) depth image; (**c**) manual annotation result; (**d**) 2D image detection result; (**e**) 3D point clouds detection result; (**f**) detection result of the proposed method.

**Figure 12:**
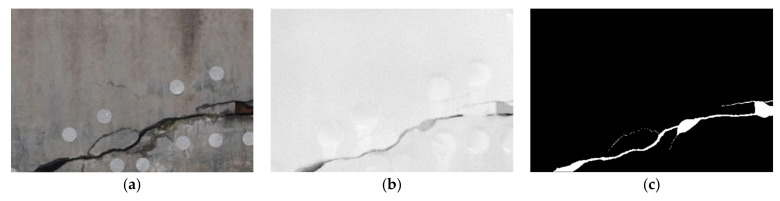

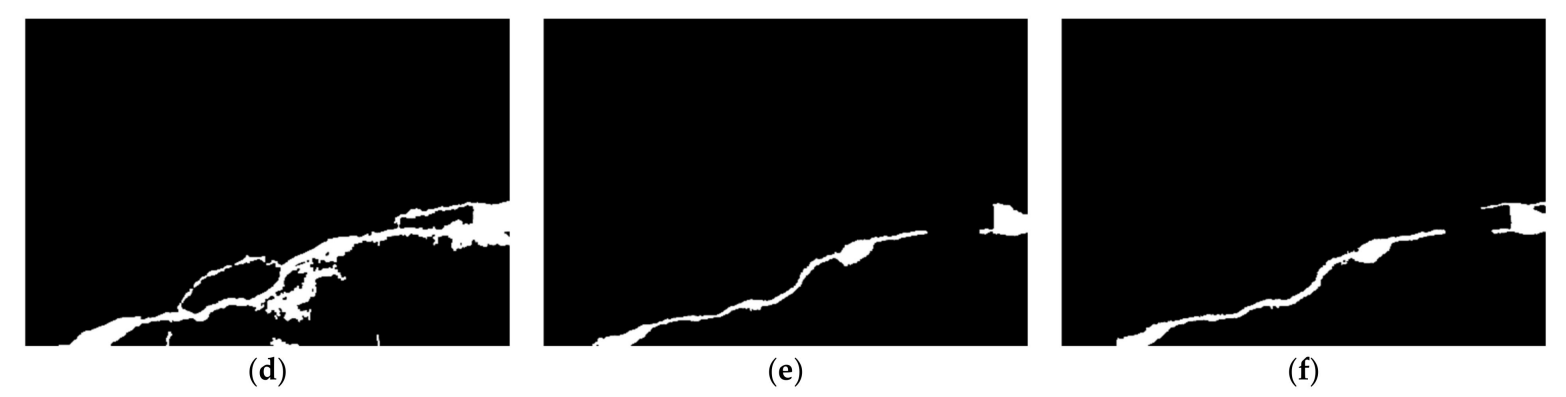
Comparison of crack detection results of different methods based on terrestrial laser point clouds: (**a**) image; (**b**) depth image; (**c**) manual annotation result; (**d**) 2D image detection result; (**e**) 3D point clouds detection result; (**f**) detection result of proposed method.

To verify the feasibility and universality of the proposed method, it was compared with a single 3D point clouds method and a single 2D-images method, respectively. All the real cracks were obtained manually for evaluation. Some of the crack detection results are as follows: Figure 11 shows the results for a group of longitudinal cracks with detection by three methods, where the point clouds data came from a handheld laser scanner. Figure 12 shows the results of a group of inclined cracks by three methods with detection, where the point clouds data came from a terrestrial laser scanner. Figure 11d and Figure 12d are the 2D image-detection results; upon comparison with the manual annotation results (Figure 11c and Figure 12c), much false detection was observed, due to there being many stains near the cracks Figure 11e and Figure 12e show the results of detection based on 3D point clouds. Although 3D point clouds are not affected by crack surface stains, the detection of small or shallow cracks is poor, resulting in fractures. Figure 11f and Figure 12f show the results of detection using the method proposed in this paper. Those shown in Figure 11f are largely consistent with the manual annotations, with no fractures in the cracks. This demonstrates that the proposed method can overcome interference from the background, with a significantly reduced rate of false detection, while still small cracks. Although the background interference was also removed as shown in Figure 12f, some cracks were not detected due to the influence of various factors, such as cracks not being obvious because of landfills, the grayscale values of stains, and background being very similar, and cracks being too small. Moreover, the point clouds of the crack in Figure 12 come from the terrestrial 3D laser scanner which is less accurate than the handheld laser scanner, so the ability to recognize the small cracks shown in Figure 12f is not as good as Figure 11f.

**Figure 13:**
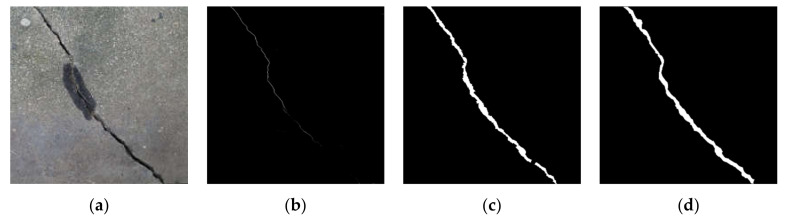
Comparison of detection results between deep learning and the proposed method: (**a**) image; (**b**) DeepCrack detection result; (**c**) detection result of proposed method; (**d**) manual annotation result.

To illustrate the superiority of the proposed method, a set of cracks was randomly selected from large experimental samples, and the deep learning crack detection algorithm called DeepCrack [34] and the method in this paper were compared. The detection results are shown in Figure 13. It can be seen from Figure 13a that there is a large area of background interference near the crack. Comparing Figure 13b,c, we can see that the method in this paper can resist noise interference and accurately extract cracks. The deep learning method recognizes large crack edge pixels as background pixels, resulting in the width of the crack detected being much smaller than the true width.

In this study, the precision, recall, and F1-measure (also known as F1 score) were used to quantitatively analyze and evaluate the performance of the proposed method. The precision measures the exactness or fidelity of detection, while the recall describes the completeness of detection. We used a common evaluation standard for the proposed method, the F1-measure (also known as F1 score), which combines precision and recall. The closer the F1 value is to 1, the better the method performs. P, R, and F1 were calculated using Equations (11)–(13), respectively. Table 3 shows the quantitative results.

(11) $$P=\frac{T_{P}}{\left(T_{P}+F_{P}\right)},$$

(12) $$R=\frac{T_{P}}{\left(T_{P}+F_{N}\right)},$$

(13) $$F1=\frac{2\times Precision\times Recall}{\left(Precision+Recall\right)},$$

where $T_{P}$ denotes true positives; that is, pixels labeled as crack pixels are correctly recognized as crack pixels; $F_{P}$ denotes false positives; that is, pixels labeled as non-crack pixels are incorrectly recognized as crack pixels; $F_{N}$ represents false negatives; that is, pixels labeled as crack pixels are incorrectly detected as non-crack pixels.

Table 4 and Table 5 show the results of detection using three methods for three types of crack. The point cloud data for the cracks shown in Table 4 were obtained by a handheld laser scanner, while the data in Table 5 were obtained by a terrestrial laser scanner. Compared with the detection method using a single 2D image, as observable from the tables, the proposed crack detection method fusing point clouds and 2D images is significantly more precise. The biggest increase in precision is seen for Crack No.3 (Table 4), by 51.8% from 38.7% to 90.5%; the recall of the single 3D point-cloud detection method fluctuates between 46.8% and 88.5% (the results for five of the six groups of data are below 80%), while that of the proposed method is consistently above 80%. According to the comprehensive evaluation index, the F1 score, the proposed fusion method is better than the single 3D-point-cloud or 2D-image methods for five of the six groups of data containing three types of crack, with F1 scores consistently above 80%. Therefore, the proposed detection method shows great performance and universality.

Table 6 shows the average detection results for all the cracks; it can be seen that the proposed method achieved an average precision of 89.0%, and a recall of 84.8%; so it is highly accurate. The average F1 score was 86.7%, which is 19.1% and 10.7% higher than the scores of the single 2D-image and 3D point-clouds methods, respectively, showing that the proposed method shows the best overall performance.

The quantitative results detected by the deep learning and the proposed method are shown in Table 7. It can be seen from the table that both accuracy and recall rate and F1 score of the proposed method are superior to the DeepCrack method.

The crack-detection results for the handheld and the terrestrial 3D laser scanners are shown in Table 8. With the single 3D method, the three groups of cracks acquired by the handheld scanner are smaller than those acquired by the terrestrial laser scanner, but the resolution of the handheld scanner (0.5 mm) is higher than that of the terrestrial (1 mm). Therefore, the average precision (90.2%), recall (76.9%), and F1 score (82.9%) of the handheld laser scanner are better than those of the terrestrial. The results obtained from fusion with high-precision point clouds were expected to be better than those obtained from fusion with low-precision point clouds, but this was not the case. This could be because the background interference in the 2D image data of three groups corresponding to the handheld laser scanner is greater than that with the terrestrial laser scanner, compromising the detection results. Comparing the detection results from different types of point cloud shows that the accuracy of the point cloud has a greater impact on the precision of the posed fusion detection method and that higher-precision point clouds can lead to better detection results. Although the point clouds collected by the handheld 3D laser scanner are more accurate, the terrestrial 3D laser scanner is far superior in terms of scanning efficiency. Therefore, the advantages of both might be harnessed by choosing between them according to the scenario: for wide cracks or large scenes with low accuracy requirements, the terrestrial 3D laser scanner could be used to obtain the point cloud data, while in the small scenes with shallow cracks and cracks buried or with high precision requirements, the handheld 3D laser scanner could be used to obtain said data. In the future, with the continuous advancement of terrestrial 3D laser-scanning technology, more accurate and faster scanning will be realized, supporting the ability of the method in this paper to detect cracks with high-precision and high-efficiency.

Pixel calibration was realized by using a circular target. Then, the corresponding true distances (GSDs) of pixels for six groups of crack images were obtained, as shown in Table 9; it can be seen that the GSDs of the three groups of images corresponding to the handheld 3D laser scanner are better than those for the terrestrial 3D laser scanner. The GSD reflects the distance between the camera and the concrete surface. In theory, the closer the camera is to the concrete surface, the higher the image resolution and the more detailed the crack information obtained. It can be seen from Table 4, Table 5 and Table 6 that the GSDs of the images of Cracks No. 1–3 higher than those of the images of Cracks No. 4–6, but the F1 value is not optimal. We speculate that this is because there were too many stains on the surface of the concrete cracks, which were themselves accurately recorded in the images with high GSDs.

Table 10 describes the maximum width error between the results obtained with the method in this paper and the manual annotations; one can see that the wider crack, the lower the relative error. For cracks with a point cloud accuracy of 0.5 mm, when the crack width is about 2 cm, the relative error for the maximum width is within 7.3%; when the crack width is less than 1 cm, the relative error exceeds 10%; For cracks with a point cloud accuracy of 1 mm, when the crack width is about 2 cm, the relative error is within 5.6%; when the crack width is greater than 5 cm, the relative error is less than 2%.

## 4. Discussion

The weighted average pixel-level image fusion method is used to fuse the depth and gray image, so the selection of the factor weighting greatly impacts the final detection accuracy. If the depth image is overweighted, it becomes difficult to effectively take advantage of the high resolution of the image, which can allow the identification of tiny cracks. By contrast, if the weight of the image is too large, then stains and uneven illumination in the image can greatly interfere with crack detection. Therefore, appropriately setting these factors according to the situation of the concrete crack and the actual measured performance of the scanner will improve the accuracy of crack detection. The weighting factors in Table 3 are the best weighting factors we chose in the experiment. The crack comes from different concrete pavements and walls in the university campus, so the type, width, and concrete conditions of the cracks are very different. Therefore, the optimal weighting factors for different fractures are different. But we did give some guiding advice. For wide cracks with greater environmental interference, we recommend that the weighting factor Kr of the depth image should be greater than 0.45; for narrow cracks with greater environmental interference, it should be greater than 0.65. In future research, we will study the influence of weight factors in depth and find a weight factor allocation method with wider applicability.

The scanner distance, incident angle, and other factors have a certain impact on the accuracy of crack detection. The existing research of Kim [20] and the findings in this paper show that the farther the terrestrial 3D laser scanner is from the concrete surface, the greater the incident angle, the sparser the point cloud obtained, and the more difficult it is to detect points inside the crack. Therefore, we recommend measuring as close as possible and orthogonal to the concrete surface, but no closer than the closest distance of the scanner. For handheld 3D lasers, repeated measurements are performed within 25–30 cm of the diseased concrete surface to obtain all the point clouds. Therefore, it is worth considering performing repeated measurements from multiple angles to obtain as much depth information as possible inside the crack. In actual engineering applications, to reduce the time cost of data acquisition, we will consider using more efficient and convenient 3D laser sensors, such as the use of Unmanned Aerial Vehicle 3D laser scanners to collect point cloud if the point cloud meets the requirements of precision and density.

The algorithm used for crack extraction has a great influence on the accuracy of detection. The proposed method, fusing point clouds and images based on an improved Otsu algorithm, not only obtains width and depth information for cracks but is also strongly resistant to interference from stains. It should be mentioned that the development of computer vision technology has made deep learning in crack-detection research a hotspot in recent years. Therefore, in the future, we will conduct in-depth research on crack detection with the fusion of point clouds and images based on deep learning.

## 5. Conclusions

To deal with the disadvantages of low accuracy in extracting concrete surface cracks based on single 3D point-cloud and 2D-image data, this paper proposes an automatic concrete crack detection method fusing 3D point clouds and 2D images based on an improved Otsu’s algorithm. Firstly, depth images from point clouds and 2D images are fused at the pixel level to generate a fused image, and then accurate crack detection is realized based on an improved Otsu method. The main contributions of this paper to the research on crack detection, regarding the proposed method, are as follows: (1) the point clouds are first processed to reduce dimensionality not only does this significantly reduces the data and necessary calculations, but mature 2D image-processing technology can be used to detect cracks from the fusion image; (2) the fusion of 3D point clouds and 2D images is realized at the pixel level. The fusion image combines the grayscale information from the high-resolution images and the depth information from the point clouds, fully harnessing the advantages and compensating for the shortcomings of both; (3) an improved Otsu crack-detection method is proposed. By dividing the image into blocks and introducing constraints related to the image sub-blocks, the error that would be introduced by the forced binarization of the image sub-blocks is avoided, thereby improving the crack detection accuracy; (4) the effects on the detection accuracy of different methods for acquiring the point clouds were studied, and methods are proposed according to the scenarios and precision requirements for crack detection. Further research will seek to improve the image segmentation algorithm.

## Figures and Tables

**Table 1:** The terrestrial laser scanner parameters.

Sensor Picture	Technical Specification	RIEGL VZ-2000
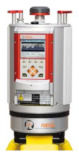	Scanning distance	2000 m
	Positioning Precision	5 mm/100 m
	Maximum angular resolution	0.0015°
	Range	Horizontal 360° (max) Vertical 100° (max)

**Table 2:** Handheld laser scanner parameters.

Sensor Picture	Technical Specification	HandySCAN700
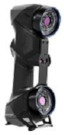	Positioning accuracy	0.03 mm
	Grid resolution	0.2 mm
	Scanning area	275 × 250 mm

**Table 3:** Relevant parameters of the proposed method.

Scanner Type	Crack	Weighting Factor		Distance	Crack Basic Information
		k_i_	k_r_		
Handheld laser	1	0.45	0.55	0.25 m	Wide crack, many stains
	2	0.26	0.74	0.25 m	Narrow crack, many stains
	3	0.35	0.65	0.25 m	Narrow crack, many stains
Terrestrial laser	4	0.80	0.20	3.15 m	Narrow crack, many stains
	5	0.45	0.55	3.01 m	Wide crack, many stains
	6	0.25	0.75	3.38 m	Narrow crack, many stains

**Table 4:** Comparison of quantitative results of crack detection (handheld laser).

Evaluation Index	Crack	2D Only	3D Only	2D and 3D
P (%)	1	58.5	98.4	97.3
	2	44.6	81.6	77.7
	3	38.7	90.6	90.5
R (%)	1	99.4	88.5	91.3
	2	87.2	74.3	82.4
	3	80.8	68.1	81.2
F1 (%)	1	73.6	93.2	94.2
	2	59.0	77.8	80.0
	3	52.4	77.7	85.6

**Table 5:** Comparison of quantitative results of crack detection (terrestrial laser).

Evaluation Index	Crack	2D Only	3D Only	2D and 3D
P (%)	4	88.4	54.6	87.9
	5	87.4	99.9	96.0
	6	42.2	85.5	84.6
R (%)	4	85.9	75.4	88.4
	5	66.6	46.8	81.7
	6	93.3	75.2	83.8
F1 (%)	4	87.1	63.4	88.1
	5	75.6	63.7	88.3
	6	58.2	80.0	84.2

**Table 6:** Comparison of the average detection results of crack detection using different methods.

Method	$\bar{P}\;(\%)$	$\bar{R}\;(\%)$	$\bar{F1}\;(\%)$
2D only	60.0	85.5	67.6
3D only	85.1	71.4	76.0
2D and 3D	89.0	84.8	86.7

**Table 7:** Comparison of crack detection results between DeepCrack and the proposed method.

Method	P (%)	R (%)	F1 (%)
Proposed method	77.7	82.4	80.0
Deep Crack	68.9	38.9	49.7

**Table 8:** Comparison of average results of crack detection by different scanners.

Scanner Type	Method	$\bar{P}\;(\%)$	$\bar{R}\;(\%)$	$\bar{F1}\;(\%)$
NO	2D only	62.7	86.4	69.1
Handheld laser	3D only	90.2	76.9	82.9
	2D and 3D	88.5	85.0	86.6
NO	2D only	80.5	82.8	79.5
Terrestrial laser	3D only	80.0	65.8	69.0
	2D and 3D	89.5	84.6	86.9

**Table 9:** Image ground sampling distance (GSD) information.

Scanner Type	Crack	Target Diameter (cm)	Target Radius (pixel)	GSD (cm/pixel)
Handheld laser	1	0.6	59.13	0.0051
	2	0.6	48.27	0.0062
	3	0.6	46.94	0.0064
Terrestrial laser	4	5	59.25	0.0422
	5	5	63.61	0.0393
	6	5	69.71	0.0359

**Table 10:** Comparison of the maximum width error between the proposed method and manual annotation.

Scanner Type	Crack	Detection Width (cm)	True Width (cm)	Absolute (Errorcm)	Relative Error (%)
Handheld laser	1	2.06	2.23	0.16	7.29
	2	1.02	0.92	0.12	10.56
	3	1.40	1.58	0.18	11.34
Terrestrial laser	4	2.66	2.70	0.04	1.56
	5	2.00	2.12	0.12	5.56
	6	5.52	5.63	0.11	1.91

## Data Availability

The data presented in this study are available on request from the corresponding author. The data are not publicly available due to privacy.
